# The white test for intraoperative screening of bile leakage: a potential trigger factor for acute pancreatitis after liver resection—a case series

**DOI:** 10.1186/s12893-021-01354-5

**Published:** 2021-10-02

**Authors:** Sophie Chopinet, Emilie Bollon, Jean-François Hak, Laurent Reydellet, Valéry Blasco, Farouk Tradi, Guillaume Louis, Emilie Grégoire, Jean Hardwigsen

**Affiliations:** 1 Department of Digestive Surgery and Liver Transplantation, Hôpital la Timone, 264 rue Saint-Pierre, 13385 Marseille Cedex 05, France; 2 Department of Anesthesiology, Hôpital la Timone, Marseille, France; 3 Department of Radiology, Hôpital de la Timone, Marseille, France; 4 Aix Marseille Univ, LIIE, Marseille, France; 5 Aix Marseille Univ, CERIMED, Marseille, France

**Keywords:** Acute pancreatitis, White test, Hepatectomy, Bile leakage, Case series

## Abstract

**Background:**

Acute pancreatitis after liver resection is a rare but serious complication, and few cases have been described in the literature. Extended lymphadenectomy, and long ischemia due to the Pringle maneuver could be responsible of post-liver resection acute pancreatitis, but the exact causes of AP after hepatectomy remain unclear.

**Cases presentation:**

We report here three cases of AP after hepatectomy and we strongly hypothesize that this is due to the bile leakage white test. 502 hepatectomy were performed at our center and 3 patients (0.6%) experienced acute pancreatitis after LR and all of these three patients underwent the white test at the end of the liver resection. None underwent additionally lymphadenectomy to the liver resection. All patient had a white-test during the liver surgery. We identified distal implantation of the cystic duct in these three patients as a potential cause for acute pancreatitis.

**Conclusion:**

The white test is useful for detection of bile leakage after liver resection, but we do not recommend a systematic use after LR, because severe acute pancreatitis can be lethal for the patient, especially in case of distal cystic implantation which may facilitate reflux in the main pancreatic duct.

## Introduction

Acute pancreatitis (AP) is a rare but serious complication after liver resection, and few cases have been described in the literature [1]. Extended lymphadenectomy [2], and long ischemia due to the Pringle maneuver could be responsible of post-liver resection acute pancreatitis, but the exact causes of AP after hepatectomy remain unclear. We report here three cases of AP after hepatectomy and we strongly hypothesize that this is due to the bile leakage white test. Bile leakage is a serious complication after liver resection (LR) rising from 3.8 to 10% [3–5], and up to 30% after major liver resection in recent series. Biliary complications after liver resection increases mortality rate, hospital stay and the development of intra-abdominal sepsis and liver failure [6]. Several studies and randomized control trial have compared the benefit of bile leakage test to reduce post LR bilious complications and several products have been compared [7]. Saline solution seems to be the less efficient agent. Blue methyl test or indocyanine green test are efficient for bile leakage detection and are easily available in all center, but the test can’t be repeated because of color contamination of the liver section. Finally, recent studies have shown the benefits of fat emulsion, generally used for parenteral nutrition, which is easily available. The white test can be repeated without contamination of the liver section after washing away with saline solution [8].

From January 2017 to November 2020, 502 hepatectomy were performed at our center and 3 patients (0.6%) experienced acute pancreatitis after LR and all of these three patients underwent the white test at the end of the liver resection.

Briefly, at the end of the hepatectomy, a cannula is inserted in the cystic duct: The fat emulsion is injected slowly after clamping the main bile duct under the cannula for screening of bile leakage on the liver section. After removing the cannula the product was let out through the cystic before closing the cystic duct to release pressure in the biliary tract. In our center, the white test is performed to detect bile leak. The white test wasn’t systematically performed at the end of each hepatectomy but only in case of high risk of bile leak at the surgeon’s discretion*.* Despite this test can be very useful to highlight bile leakage, we deplored adverse events that have never been described in the literature.

We report here, a three-cases series of severe acute pancreatitis after liver resection. None underwent additionally lymphadenectomy to the liver resection. The three patients had a white-test during the liver surgery and we identified distal implantation of the cystic duct in these three patients.

## Case A

**Fig. 1:**
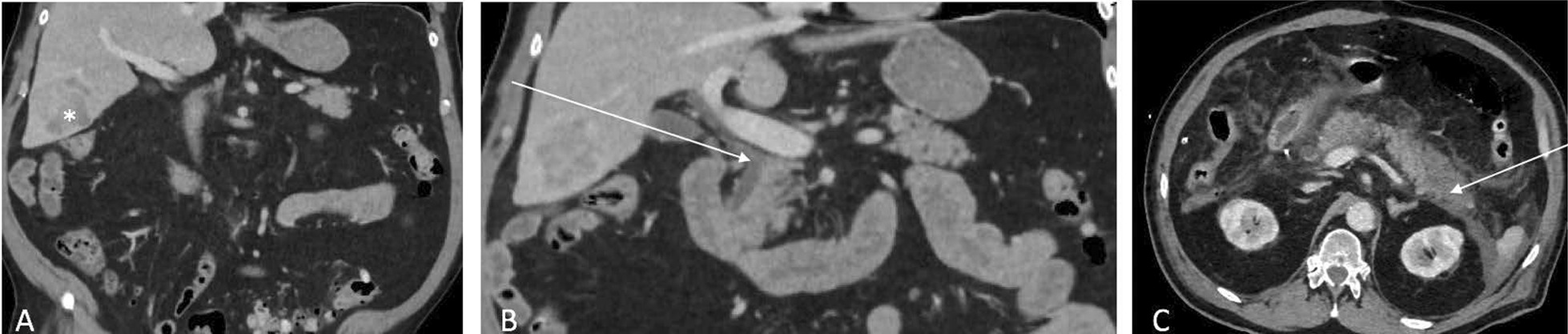
Patient A. **A** Preoperative CT showing HCC in segment VI (white asterisk). **B** Preoperative CT, distal implantation of the cystic duct (white arrow). **C** CT scan at POD 1 showing necrosis of the tail of the pancreas t

Patient A, a 72-year-old man, underwent a bisegmentectomy V–VI for hepatocellular carcinoma on NASH in November 2020. His comorbidities were high blood pressure, diabetes mellitus, and atrial fibrillation under treatments of anticoagulants. The review of his medical history showed that the white test was a trigger factor for post-hepatectomy pancreatitis. Upon admission, a bisegmentectomy was carried out, an intermittent portal triad clamping (10/5), the pringle maneuver was performed, the total warm ischemia time was 47 min. No complications occurred. The amount of blood loss was estimated at 300 cc. However, at the end of the parenchymal transection, bile leakage was suspected and a bile leakage test or white test was performed. A cannula was introduced in the cystic duct, and fat emulsion was injected slowly. The bile duct was clamped at the upper border of the pancreas to put the intrahepatic bile duct on pressure and to facilitate the visualization of the bile leakage. No bile leakage was highlighted with the white test. No complications occurred during the following 24 h. The patient was discharge from the intensive care unit at 48 h. Rapidly, his respiratory condition deteriorates, with hemodynamic failure, metabolic acidosis, and coagulopathy. A CT was performed at 72H postoperatively, and showed a necrotic pancreatitis with a necrosis of 40% of the body and tail of the pancreas (Fig. 1). The patient was admitted at the ICU, and his condition continued to deteriorate and died from multi-organ failure.

## Case B

Patient B, a 66-year-old man, who underwent right hepatectomy in January 2017 for HCC on F2 liver fibrosis post hepatitis C infection experienced post liver resection pancreatitis. Review of this case showed the white test as the trigger factor of pancreatitis. During the surgery, portal intermittent clamping was performed (15/5) and the whole warm ischemic time was 48 min. After the surgery, several cofounding factors have hampered the diagnosis of pancreatitis. Postoperative bleeding occurred at postoperative day 5. A diaphragmatic artery has been embolized. An abdominal compartment syndrome occurred 2 days after the radiologic embolization due to the amount of hemoperitoneum. An open peritoneal toilet was performed with copious volumes of warm normal saline. The patient condition rapidly improved until postoperative day 14, when he experienced severe sepsis. The abdominal CT showed a large collection in the resection area and peri-pancreatic collection. A bilioma was suspected and an open surgical drainage and peritoneal toilet was performed. No bile leakage and no bile fluid were identified, but there was multiple septic collections and cystosteatotic nodes. Intra-peritoneal fluid dosage revealed high amount of Amylase > 4000 UI/L and Lipase > 4000 UI/L and pancreatitis was diagnosed. The clinical condition improved and the patient discharged from the hospital at day 45. A Cholangio-pancreato-MRI was performed at postoperative day 90 and showed a pseudocyst in the uncus of the pancreas. No long-term complications, and no HCC recurrence during the follow-up. The follow-up time was 40 months.

## Case C

**Fig. 2:**
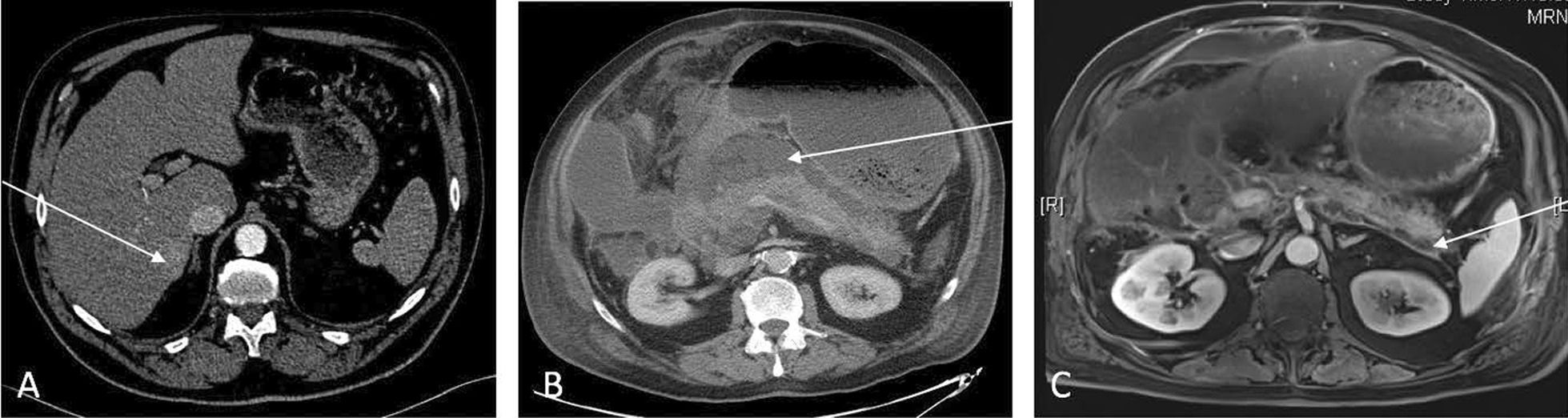
Patient C. **A** Preoperative CT showing HCC in segment VI. **B** Postoperative CT scan at day 14 showing acute pancreatitis with abdominal collections (white arrow). **C** MRI, T1-weight, at 1 month after acute pancreatitis showing regression of the abdominal collections (white arrow)

Patient C, a 65-years-old man, with high blood pressure, hypercholesterolemia, and chronic alcohol consumption comorbidities underwent posterior sectoriectomy for HCC on October 2017. Preoperative blood test showed: platelets: 160 G/L, Prothrombin time: 100%, AFP: 8.1 ng/mL, and normal liver test. An Intermittent pedicule clamping (10/5) was performed, the total warm ischemic time was 34 min. A bile leakage white test was performed at the end of the liver resection. No bile leakage was identified. One hour after the surgery, hemorrhage in the abdominal drain occurred and an evacuation of the hemoperitoneum has been performed. His clinical condition improved, and the patient was discharged from the hospital at POD 7. At POD 14, the patient experienced abdominal sepsis, fever and abdominal pain. An abdominal CT showed: severe pancreatitis with walled-off pancreatic necrosis (Fig. 2). External radiologic tube was placed to drain walled-off pancreatic necrosis. Despite efficient drainage, his clinical condition was deteriorating and a reintervention was performed to drain septic peri-pancreatic collections. Then, his clinical condition rapidly improved and the patient was discharged at POD 35. No long-term complications, and no recurrence occurred during the follow-up during the 36 months.

Table 1 summarize the patient’s parameters.

**Table 1:** Characteristics of the three patients

Patient	White test	Acute pancreatitis grade (CTSI)	Postoperative day of acute pancreatitis onset	Type of liver resection	Intermittent pedicule clamping (duration)	Hospital stay (day)
Patient A	Yes	8	D1	Bisegmentectomy V–VI	Yes (47 min)	4
Patient B	Yes	4	D10	Right hepatectomy	Yes (48 min)	45
Patient C	Yes	6	D14	Posterior sectoriectomy	Yes (34 min)	35

An informed consent was received from all the patients prior to the study and, an ethical approval was obtained from the Sud Mediterranean Hospital Ethical committee (Sainte-Marguerite hospital, Marseille).

## Discussion

The aim of this three-cases series is to notify hepatobiliary surgeon about the risk of acute pancreatitis after liver resection due to the white test, especially when the cystic duct is implanted distally. Acute pancreatitis (AP) is a rare but serious complication after liver resection. Sathasivam et al., described a three fatal case series of acute pancreatitis after LR with no obvious cause identified. One case of AP was described after adult-to-adult living donor liver transplantation [9]. Other studies reported an AP after extended lymphadenectomy [2]. None of the patients in our series had extended lymphadenectomy associated to liver resection. We have hypothesized that reflux in the main pancreatic duct (MPD) is favored by a distal implantation of the cystic duct. In case of distal implantation of cystic duct clamping of the pedicle to put pressure on the main bile duct remains hard to perform. Data regarding the implantation of the cystic duct were not available for all patients who underwent hepatectomy at our center. But, all of the three patients had a distal cystic duct implantation. In this series of cases, the white-test was performed with a cannula inserted in the cystic duct, and the remaining hepatic duct is usually closed during the liver transection.

The white test is largely recommended to reduce the bile leakage rate during a hepatectomy (Table 2). Linke et al. [8] in a meta-analysis described that injection of the fat emulsion in the cystic duct, or in the right or left hepatic duct, during hepatectomy reduce the incidence of biliary leakage but the authors suggested to performed a randomized controlled trial in hepatic surgery of white test vs no test after hepatic surgery.

**Table 2:** Characteristics of the studies with intraoperative bile leakage white test

Authors	Year	Study design	Sample	comparison	Country
Li et al	2009	Cohort	63 vs 74	WT vs No test	Germany
Liu et al	2012	RCT	53 vs 54	WT vs No test	China
Leelawat et al	2012	Cohort	30	WT + saline test	Thailand
Linke et al	2015	Cohort	32 vs 93	WT vs No test	Germany

In living donor hepatectomies, intraoperative cholangiography and methylene blue test is usually performed to lead to higher intraoperative detection of bile leak [10], however no adverse events occurred.

This case series has several limitations, as it is difficult to prove that acute pancreatitis is directly related to the injection of the white test and not to any other non-specific cause after liver surgery. Regarding cases B and C, several factors can be confusing. In case B, an embolization for hemorrhage from a diaphragmatic artery was performed, but the embolization was selective and distant from any branches vascularizing the pancreas. In case C, the patient had a history of hypercholesterolemia treated with a statin drug, and a chronic consumption of alcohol that had been weaned off 1 month prior to the surgery. In this study, acute pancreatitis has been proven by biological tests and confirmed on imaging exams. The only common elements between these three cases were the use of the white test during hepatectomy. In conclusion the white test is useful for detection of bile leakage, but could have adverse effects and lead to severe acute pancreatitis, especially in cases of distal implantation of the cystic duct.

## Data Availability

Not applicable.

## Abbreviations

AP: Acute pancreatitis
LR: Liver resection
MPD: Main pancreatic duct
